# 811 A Dermal Scaffolds Regulate Early Vascularization to Promote Wound Healing by Co-expressing VEGF and aFGF

**DOI:** 10.1093/jbcr/iraf019.342

**Published:** 2025-04-01

**Authors:** Wang Jialiang, wang Xingang

**Affiliations:** Department of Burns & Wound Care Center, Second Affiliated Hospital of Zhejiang University; Department of Burns & Wound Care Center, Second Affiliated Hospital of Zhejiang University

## Abstract

**Introduction:**

In contemporary medical practice, deep skin defects resulting from severe burns, mechanical injuries and other acute or chronic traumas pose challenges to heal. Over the past few decades, the artificially synthesized tissue-engineered skin substitutes have been developed. However, the clinical success of these dermal substitutes is often undermined by their limited in vivo angiogenesis rates. The urgent need to enhance the vascularization of dermal substitutes and improve their repair efficiency and effectiveness presents a significant challenge. To further enhance the efficiency of vascularization, we encapsulated plasmid DNA nanocomposite particles (pDNA NPs) of VEGF (vascular endothelial growth factor) and aFGF (acidic fibroblast growth factor) within the PLGA knitted mesh-reinforced collagen/chitosan scaffolds (referred to as DGAS-M), thereby developing a scaffold imbued with dual gene activities.

**Methods:**

In this study, we conducted a comprehensive evaluation of the effects on the proliferation of human fibroblasts, angiogenesis, and the synthesis and secretion of growth factors in umbilical vein endothelial cells of DGAS-M in vitro. Then, we employed a rat subcutaneous implantation model to select optimal gene loading. Furthermore, we evaluated the ability to promote wound healing by using rat model featuring full-thickness.

**Results:**

In this study, we developed DGAS-M co-expressing VEGF and aFGF. DGAS-M shows effects on the proliferation of human fibroblasts, angiogenesis, and the synthesis and secretion of growth factors in umbilical vein endothelial cells in vitro. Among the examined dosages, the DGASs-M loaded with 15 μg/unit pDNA NPs exhibited a significantly vascularization during the early stages of implantation. Furthermore, in a rat model featuring full-thickness skin defects, DGAS-M significantly enhanced the survival rate of autologous split-thickness skin grafts during the first 14 days post-surgery by shortening the vascularization time and reducing the release of inflammatory factors on wound.

**Conclusions:**

In this experiment, DGAS-M exhibit the capacity to enhance the proliferation of human fibroblasts and stimulate angiogenesis in umbilical vein endothelial cells, while also boosting the secretion of VEGF and aFGF. Our findings also demonstrated DGAS-M markedly reduced the vascularization time in the wound, diminished the release of inflammatory factors, and consequently facilitated defect healing while enhancing overall healing quality.

**Applicability of Research to Practice:**

This approach holds the potential to facilitate rapid vascularization of dermal substitutes in full-thickness skin defects and enable the swift integration of split-thickness skin grafts transplanted via a single-step procedure. Therefore, the dermal scaffolds we have constructed will contribute to the deep skin defects resulting from severe burns, mechanical injuries and other acute or chronic traumas pose challenges to heal.

**Funding for the Study:**

This work was supported by grants from the National Key R&D Program of China (2022YFC2403104).

